# Heat-Induced Proteotoxic Stress Response in Placenta-Derived Stem Cells (PDSCs) Is Mediated through HSPA1A and HSPA1B with a Potential Higher Role for HSPA1B

**DOI:** 10.3390/cimb44100324

**Published:** 2022-10-10

**Authors:** Bothina Mohammed Alharbi, Aisha Bugshan, Azhaar Almozel, Reem Alenzi, Abderrezak Bouchama, Tanvir Khatlani, Sameer Mohammad, Shuja Shafi Malik

**Affiliations:** 1 Experimental Medicine Department, King Abdullah International Medical Research Center, King Saud bin Abdulaziz University for Health Sciences, Ministry of National Guard Health Affairs, Riyadh 11426, Saudi Arabia; 2 Stem Cells Unit, Blood and Cancer Research Department, King Abdullah International Medical Research Center, King Saud bin Abdulaziz University for Health Sciences, Ministry of National Guard Health Affairs, Riyadh 11426, Saudi Arabia

**Keywords:** proteostasis, heat shock, chaperones, HSPA1B, HSPA1A, stress response, placenta-derived stem cells, placenta, stem cells

## Abstract

Placenta-derived stem cells (PDSCs), due to unique traits such as mesenchymal and embryonic characteristics and the absence of ethical constraints, are in a clinically and therapeutically advantageous position. To aid in stemness maintenance, counter pathophysiological stresses, and withstand post-differentiation challenges, stem cells require elevated protein synthesis and consequently augmented proteostasis. Stem cells exhibit source-specific proteostasis traits, making it imperative to study them individually from different sources. These studies have implications for understanding stem cell biology and exploitation in the augmentation of therapeutic applications. Here, we aim to identify the primary determinants of proteotoxic stress response in PDSCs. We generated heat-induced dose-responsive proteotoxic stress models of three stem cell types: placental origin cells, the placenta-derived mesenchymal stem cells (*pMSCs*), maternal origin cells, the decidua parietalis mesenchymal stem cells (*DPMSCs*), and the maternal–fetal interface cells, decidua basalis mesenchymal stem cells (*DBMSCs*), and measured stress induction through biochemical and cell proliferation assays. RT-PCR array analysis of 84 genes involved in protein folding and protein quality control led to the identification of Hsp70 members HSPA1A and HSPA1B as the prominent ones among 17 significantly expressed genes and with further analysis at the protein level through Western blotting. A kinetic analysis of HSPA1A and HSPA1B gene and protein expression allowed a time series evaluation of stress response. As identified by protein expression, an active stress response is in play even at 24 h. More prominent differences in expression between the two homologs are detected at the translational level, alluding to a potential higher requirement for HSPA1B during proteotoxic stress response in PDSCs.

## 1. Introduction

Placenta-derived stem cells (PDSCs) are a type of mesenchymal stem cells (MSCs) that harbor both embryonic and mesenchymal stem cell characteristics while possessing differentiating advantages of immune tolerance such as non-carcinogenic status [1,2,3]. Despite having a mesodermal phenotype, PDSCs still display broad differentiation potential and can differentiate into all embryonic germ layers [2,3,4]. The dispensability of the placenta post-delivery resolves ethical concerns that are integral to embryonic stem cells [1,3,5] and allows the large-scale availability of placenta-derived tissues and stem-cell derivatives without the need for subjecting donors to invasive surgical procedures [1,4]. These features make PDSCs an attractive alternative in cell replacement therapies and regenerative medicine.

PDSCs, similar to other stem cells, require elevated protein synthesis emanating from their requirements to maintain stemness and differentiation potential [6,7,8,9]. The requirement for dynamic protein synthesis is compounded by their ability to sense and respond to varying conditions and stresses from different physiological and cell-habitat sources [7,10]. Consequently, the necessity for constant protein production creates a state of proteome level stress. This exerts additional demand on the proteome regulating machinery to ensure proteome adjustment to endogenous needs of the cells in a spatio-temporal manner. The protein homeostasis (proteostasis) network in the cells coordinates the proteome balance by regulating all steps of protein life cycle: synthesis, folding, conformational maintenance, and degradation [6,9,11,12]. There potentially is a close connection between proteostasis and stem cell function, highlighting the presence of stem cell intrinsic proteostasis mechanisms and their tight coupling to cellular properties and functions [7,8,13,14]. The determining goal for proteostasis is to ensure the operational levels of proteins in their native conformations, while simultaneously reducing the presence of deleterious products such as aggregates [6,12,15]. Proteostasis, as a result, is critically dependent upon a complex network of proteins called chaperones which function in different stages of the protein life cycle [11,12,14].

Furthermore, 70-kDa heat shock proteins (Hsp70s) are a ubiquitous group of chaperones that assist in various processes, including folding of nascent proteins, refolding of aggregated proteins, protein trafficking, and degradation of irreversible aggregates [16,17]. Similar to other chaperones, Hsp70s display little specificity, but form a critical component of the protein-folding machinery due to a high degree of adaptation in their functional properties, largely attributed to their interactions with other function complementing proteins [12,18,19]. As a result of these properties and their association with different phases of the protein life cycle, the stress inducibility of Hsp70s becomes a crucial factor in the maintenance of cellular health [14,20]. Within the Hsp70 family, HSPA1A and HSPA1B are the closest ones in differing only by two amino acids, but having their own defined and diverse roles, for example, during cancer [17,21]. The fetal membranes forming a specialized interface between mother and fetus are rich in different types of cells, including mesenchymal cells. With the growth of the fetus, these membranes expand, spreading these cells to different regions of the placenta: placental origin cells are placenta-derived mesenchymal stem cells *pMSCs*, maternal origin cells are decidua parietalis mesenchymal stem cells *DPMSCs*, and those from the maternal–fetal interface are decidua basalis mesenchymal stem cells, *DBMSCs* [22,23,24,25]. Despite the common placental origin, their different niches, and the specificity of proteostasis mechanisms to stem cell types warrant their study individually. Here, we report the quantitative gene expression analysis of the protein-folding pathway in a heat-induced proteotoxic stress model in these three placenta-derived stem cells. The proteotoxic stress models generated here potentially mimic the stress conditions that stem cells experience during their life cycle and differentiation. We intended to induce proteotoxic stress in the cells in a controlled manner to identify the main protagonists of the proteotoxic stress response. Biological systems are adapted to grow at optimum temperatures which largely reflect the structural and functional stability limits of their proteins [20]; consequently, we used heat shock as proteotoxic stress inducer. We screened these stress-induced cells in an 84-gene RT-PCR array for overexpression of genes implicated in protein folding and heat shock response regulation. We follow this with a detailed analysis of the stress response time-series gene and protein expression of Hsp70 members HSPA1A and HSPA1B, the two top-hit genes from the RT-PCR array analysis. An active stress response is in play even at 24 h, with prominent differences in HSPA1A and HSPA1B expression detected at the translational level, alluding to a potential higher requirement for HSPA1B. We hope the outcome of this study will shed significant and new light on an essential component of the placenta-derived stem cells proteostasis network and help develop our understanding of their biology and identity. This knowledge, in turn, can help manipulate these factors [26,27,28] to enhance the utilization of these stem cells in cell therapy and other clinical applications.

## 2. Materials and Methods

### 2.1. Isolation and Culture of Placenta-Derived Stem Cells

The three placenta-derived stem cells reside in different zones of placenta and the cell cultures of these three cell types were achieved utilizing the already established protocols for their isolation, characterization, and sub-culturing: pMSCs [22], DPMSCs [24], DBMSCs [23]. The placentae were obtained from uncomplicated pregnancies following normal vaginal delivery (38–40 weeks’ gestation) and were utilized within 2–3 h of delivery. For *DBMSCs*, 10 g of the decidua tissue was dissected from the maternal surface of placenta and washed with phosphate buffered saline (PBS, pH 7.4) to remove excess blood. The tissue was then finely minced and washed with PBS until the fluid was free of blood. After centrifugation at 300× *g* for 5 min, the tissue pellet was digested using 0.3% collagenase type I (Life Technologies, Grand Island, NY, USA) diluted in PBS, 100 μg/mL streptomycin, 100 U/mL penicillin, and 271 units/mL DNase I (Life Technologies, Grand Island, NY, USA) at 37 °C for 1 h. The mixture was then filtered with a 100 μm nylon filter (Becton Dickinson, Franklin Lakes, NJ, USA) and centrifuged. The red blood cells were lysed by incubating the suspension with red blood cell lysing buffer (#sc-3621, FCM Lysing solution, Santa Cruz, CA, USA) for 45 min at room temperature (RT). After centrifugation of the cell suspension, the cells were washed and cultured at 37 °C in a humidified atmosphere containing 5% CO_2_ and 95% air, in complete DBMSC culture medium containing Dulbecco’s Modified Eagle Medium nutrient mixture F-12 (DMEM-F12), 10% Mesenchymal Stem Cell Certified Fetal Bovine Serum (MSCFBS, Life Technologies, Grand Island, NY, USA), 100 μg/mL l-glutamate, 100 μg/mL streptomycin, and 100 U/mL penicillin. For *DPMSCs*, choriodecidua was manually separated from the amnion and washed thoroughly with phosphate buffered saline (PBS, pH 7.4). The tissues (10 g) were extensively washed with PBS, minced, and then placed in prewarmed Hanks balanced salt solution (HBSS, Life Technologies, Grand Island, NY, USA) at 37 °C. The tissue suspension was then centrifuged at 1000× *g* for 5 min at room temperature (RT) and the supernatant was discarded. Red blood cells were then lysed using red blood cell lysing buffer (#sc-3621, FCM Lysing solution, Santa Cruz, CA, USA) for 45 min at RT. After centrifugation, the tissue pellet was incubated in a digestion solution containing 0.05% trypsin-EDTA (Life Technologies, Grand Island, NY, USA), 271 unit/mL DNase I (Life Technologies, Grand Island, NY, USA), 100 µg/mL streptomycin, and 100 U/mL penicillin at 37 °C in a water bath for 10 min, then washed twice in 50% fetal bovine serum (FBS)/Dulbecco’s Modified Eagle Medium Nutrient Mixture F-12 (DMEM-F12) for 10 min and centrifuged after each wash. The resulting cell pellet was resuspended in complete culture medium [DMEM-F12 containing 20% Mesenchymal Stem Cell Certified fetal bovine serum, 100 µg/mL l-glutamate, 100 µg/mL streptomycin and 100 U/mL Penicillin] and cultured—at 37 °C in a humidified atmosphere containing 5% CO_2_ and 95% air (Cell culture incubator) (passage P0). *pMSCs* were isolated by dissection of placental tissues after removing the superficial layer of maternal decidua on the maternal side of the placenta. This was followed by cutting of underlying fetal chorionic villi into small pieces of approximately 40 mg total wet weight and removal of any residual decidual tissue. After extensively washing with PBS, the tissue was incubated with TrypLE express Digestion Solution with gentle rotation overnight at 4 °C. After this, the tissue was washed with sterile PBS and allowed to adhere to the bottom of the well in 6-well plates at 37 °C in a humidified atmosphere containing 5% CO_2_ (a cell culture incubator). To this, complete cell culture medium and culture tissues at 37 °C were gently added in a cell culture incubator with change of media every 72 h. On day 14, cells that had migrated out from the cut ends of the tissues were harvested with TrypLE™ Express detachment solution. After this, cells were seeded at a density of 1 × 105 cells in 75 cm^2^ flask. After harvesting the cells are at passage zero, they were used for subsequent experiments at passage two.

### 2.2. Heat Stress Experimentation

Heat stress in cells was induced by transferring ~60% confluent cells grown at 37 °C to the temperature to be tested. At the zero time-point, all dishes (with 60–65% confluent cells) were transferred to the heat stress temperature other than the control that continued to grow at 37 °C. The exposure temperatures tested were 42, 44, and 46 °C and the cells were initially exposed for 1, 2, and 3 h. Cell physiology post-stress, i.e., during recovery phase, was monitored by transferring cells back to ambient growth temperature, i.e., 37 °C, and the cells were harvested at each time point and processed according to the next planned downstream step. Briefly, heat-stressed and control cells were washed twice with PBS and trypsinized. The cells were centrifuged at 5000 rpm for 5 min and the supernatant was discarded, and the pellets were frozen for subsequent analyses.

### 2.3. RNA Isolation and cDNA Synthesis

Total RNA was isolated from frozen cell pellets using mini RNeasy Mini Kit (Qiagen, Germantown, MD, USA). RNA integrity and yield was analyzed and quantified using the Nano Drop (Thermo Fischer, Wilmington, DE, USA). An amount of 2 µg of the total RNA was transcribed into cDNA for all gene arrays and RT-PCR experiments, using FastLane Cell cDNA Kit (Qiagen, Germantown, MD, USA).

### 2.4. Protein Aggregation Assay

Protein aggregation as an indicator of cellular stress was measured in the heat-stressed cells and tracked during the recovery phase using the 96-well Protein Aggregation Assay Kit (Cat No: ab234048) supplied by Abcam. This assay relies on binding of a fluorescent probe to the aggregated proteins involving excitation at 440 nm and emission at 500 nm. A total of 50–100 µg protein was required per well, and the samples were read in triplicates during each run. Cells from three experiments were assayed for presence of aggregates. The protein extraction for aggregation assay was accomplished through freezing and thawing cycles to avoid interference from the detergents present in the standard cell lysis buffers. The data were analyzed with reference to the control samples grown at 37 °C and represented at percentage increase in fluorescence.

### 2.5. Cell Proliferation Assays

The heat-stress models were evaluated at cellular level for impact of heat-stress on viability and proliferation. The xCELLigence Real-Time Cell Analyser (RTCA-DP version; Roche Diagnostics, Mannheim, Germany) continuously monitors cellular adherence recording label-free changes in electrical impedance [29]. This system uses an electronic readout called *impedance* (resistance to alternating current) used to express the impeded electron flow generated by disruption of interaction between electrodes and bulk solution and is stated as arbitrary units called Cell Index (CI), the magnitude of which is dependent on cell number, morphology, size, and on the strength of cell attachment to the plate surface. An initial titration of different cell densities (5, 10, and 20,000 cells/well) was performed and 10,000 cells was found to be the ideal cell density for seeding. Cells growing in the cell culture dishes were trypsinized, counted using trypan blue, and then resuspended in the culture medium. Wells of the E-16 plates were equilibrated with the culture media and background measurements were taken. Cells were then plated at density of 10,000 cells/well in fresh medium to a final volume of 200 µL and incubated for 30 min at 37 °C and 5% CO_2_ in the RTCA cradle. The impedance signals were recorded for every 10 min over a period of 72 h in control cells grown at 37 °C and heat exposed cells.

### 2.6. Gene Arrays and RT-PCR

For gene expression analysis, we initially monitored the expression of individual Hsp70 gene followed by a comprehensive analysis of 84 heat shock protein genes through use of PCR array RT^2^ Profiler™ PCR Array Human Heat Shock Proteins & Chaperones (Cat. No: 330231PAHS-076ZA Qiagen, Germantown, MD, USA) (See Table S1 for gene list). The genes that are part of this PCR Array are HSP90 (81 to 99 kD), HSP70 (65 to 80 kD), HSP60 (55 to 64 kD), HSP40 (35 to 54 kD), small HSPs (=34 kD), and other chaperone cofactors that are directly involved in different aspects of protein folding process. This array helps to simultaneously profile the expression of 84 heat shock protein genes, in addition offering the capacity to simultaneously evaluate results utilizing five endogenous controls. In our data analysis, we used two controls, β-actin and GAPDH.

The primer sequences for Hsp70 gene used for results reported in Section 3.1 were obtained from the Harvard Primer Bank repository [30]. The primers used were as follows: HSPA1B, 5′-GCGAGGCGGACAAGAAGAA-3′ (forward) and 5′-GATGGGGTTACACACCTGCT-3′ (reverse); GAPDH, 5′-GGAGCGAGATCCCTCCAAAAT-3′ (forward), and 5′-GGCTGTTGTCATACTTCTCATGG-3′ (reverse). Quantitative measurement of gene expression for individual gene and those in array was carried out with RT-polymerase chain reaction using Platinum PCR SuperMix (Thermo Fisher Scientific, Baltics, Vilnius, Lithuania) in triplicate with SYBR Green PCR Mix (Qiagen, Germantown, MD, USA).

### 2.7. Protein Extraction and Concentration Determination

Protein extraction from frozen and stored cell pellets was performed by use of RIPA Lysis and Extraction Buffer (Thermo Scientific™, Rockford, IL, USA, Cat.No: 89900). The cells were thoroughly resuspended in RIPA buffer, vortexed, and incubated on ice for 30 min prior to centrifuging at 10,000× *g* for 20–30 min at 4 °C to separate the cell debris. Protein quantification was accomplished by use of Thermo Scientific™, Rockford, IL, USA, Pierce™ BCA Protein Assay Kit. Protein concentrations were determined using a 96-well format and evaluated with reference to a standard such as bovine serum albumin (BSA).

### 2.8. Immunoblotting

Equal quantities of extracted proteins were run on 10% Sodium Dodecyl Sulfate Polyacrylamide gel and subsequently transferred onto a nitrocellulose membrane using Mini transblot system (Bio-Rad, Hercules, CA, USA). Due to the very high homology between HSPA1A and HSPA1B, their detection and differentiation requires greater amount of caution. We used primary antibodies anti-HSPA1A (Invitrogen™, Rockford, IL, USA: PA5-28003) and anti-HSPA1B (Invitrogen™, Rockford, IL, USA: PA5-28369). The immunogen for anti-HSPA1A is the region within amino acids 308 and 641 of Human HSP70 1A and for anti-HSPA1B a region within amino acids 377 and 569 of Human HSP70 1B. Presumptively, these antibodies differentiate on basis of a single amino acid difference at position 499 reported in some studies [31]. These antibodies are further validated by advanced validation methods to ensure their specificity. Anti-HSPA1B (Invitrogen™, Rockford, IL, USA: PA5-28369) has been utilized for specific detection previously [32]. In our case, we found that the amount of input protein and antibody dilution as well was critical to specific protein detection. For HSPA1A, 7 μg total protein was utilized in case of DBMSCs and DPMSCs, whereas for pMSCs, it was 15 μg. For HSPA1B, it was 10 μg total protein for DBMSCs and DPMSCs and 20 μg for pMSCs. The immunoblotting was performed by standard procedure that includes probing of membranes with primary antibodies overnight at 4 °C followed by probing with Specific horseradish peroxidase (HRP)-conjugated secondary antibodies. The blots were visualized using SuperSignal™ West Femto Chemiluminescent Substrate (Thermo Fisher Scientific, Waltham, MA, USA) in a ChemiDoc visualization system (Bio-Rad, Hercules, CA, USA). Densitometry of the bands was performed by the image analyzing software ImageJ [33] and they were normalized by protein levels of GAPDH.

### 2.9. Expression Data Analysis

All the data are presented as mean ± standard deviation (SD). Statistical differences among different groups were evaluated by one-way analysis of variance (ANOVA) using GraphPad Prism 7.0 software (GraphPad Software Inc., La Jolla, CA, USA). *p*-values < 0.05 are considered as statistically significant.

### 2.10. Expression Kinetics Analysis

**Figure 1:**
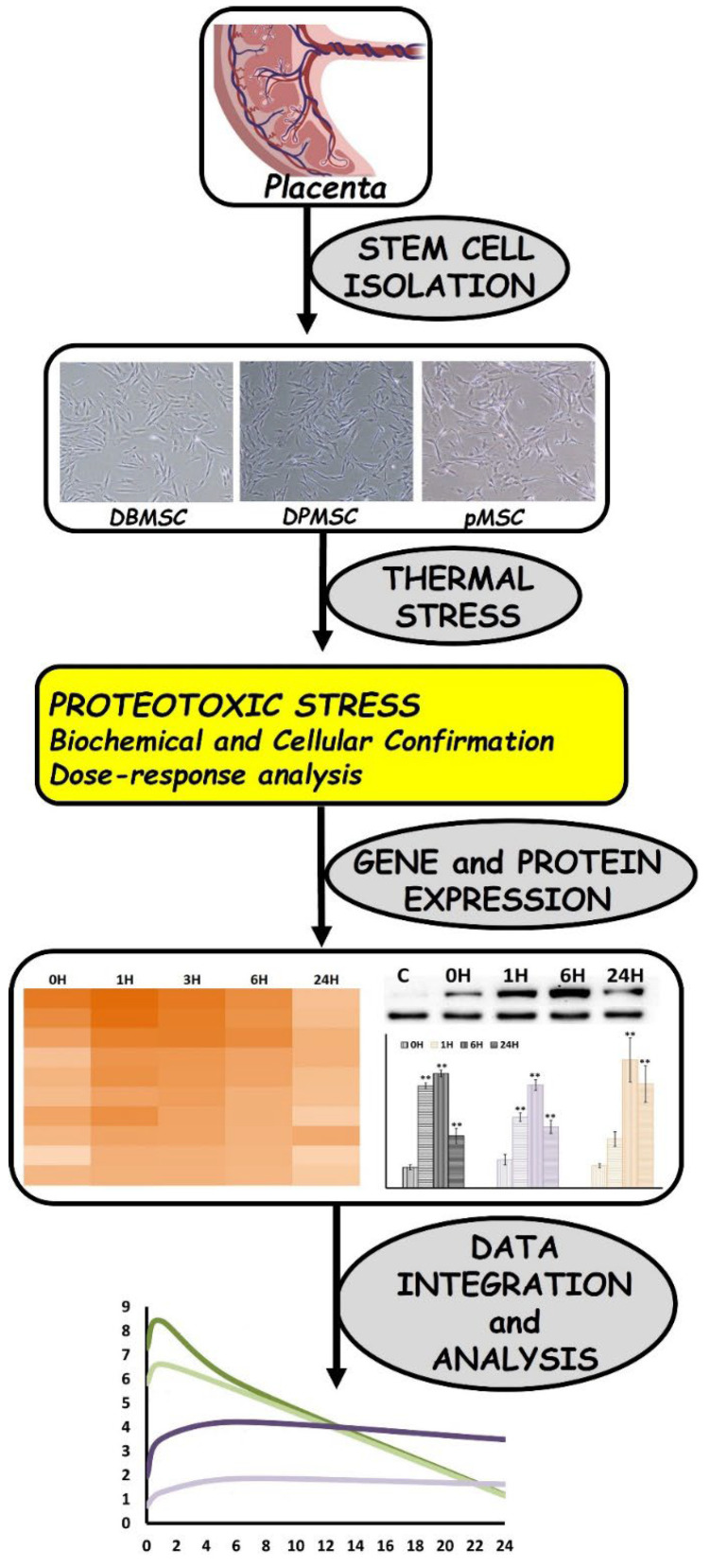
Schematic representation of the research design and methodology. Briefly, the three types of placental origin stem cells—the DBMSCs, DPMSCs, and pMSCs—were isolated from full-term pregnancy placentae. Dose-responsive proteotoxic stress models were developed through induction of thermal stress and validated through biochemical and cell-based assays. Changes in gene expression were analyzed via RT-PCR array, whereas protein expression was monitored through immunoblotting. Kinetic analysis of gene and protein expression was performed to undertake time-series evaluation of stress response. Protein expression is reported to be statistically significant ** at *p*-values < 0.05.

Expression Kinetics Analysis was performed by fitting the averaged Log_2_ fold-change in expression in Heatmap plots or Smoothened scatterplots. Wherever required, ratios were calculated utilizing the actual fold change expression data. Figure 1 depicts a schematic representation of the adapted research methodology.

## 3. Results

### 3.1. Cellular Models of Proteotoxic Stress

For screening the effective heat-stress conditions, we opted for relatively higher temperatures as compared to the optimum temperature of 37 °C. There are two reasons for this. Stem cells, by virtue of their inherent biological traits, harbor elevated proteostasis modulation and elevated levels of heat shock proteins [8,10]. Additionally, PDSCs are exposed to physiological oxidative stress within placental ecosystem that accords differential stress ameliorating capacity to these stem cells [22,23,24]. Exposure temperatures [ET] of 42, 44, and 46 °C and exposure durations [ED] of 1, 2, and 3 h formed part of the heat-stress induction protocol in decidua basalis mesenchymal stem cells (DBMSCs), decidua parietalis mesenchymal stem cells (DPMSCs), and placenta-derived mesenchymal stem cells (pMSCs). Exposing cells to heat-stress at 46 °C resulted in morphological changes and cell death, as measured by trypan blue dye uptake, and the three cells were tolerant to 44 °C, whereas DBMSCs and DPMSCs remained morphologically unaltered for up to 3 h, pMSCs stayed stable for only up to 2 h of exposure (Figure S1). Post-stress, the cells were allowed to recover at 37 °C and samples were harvested at multiple time points up to 24 h. This led to the development of a ‘time-course’ approach to study the stress response.

**Figure 2:**
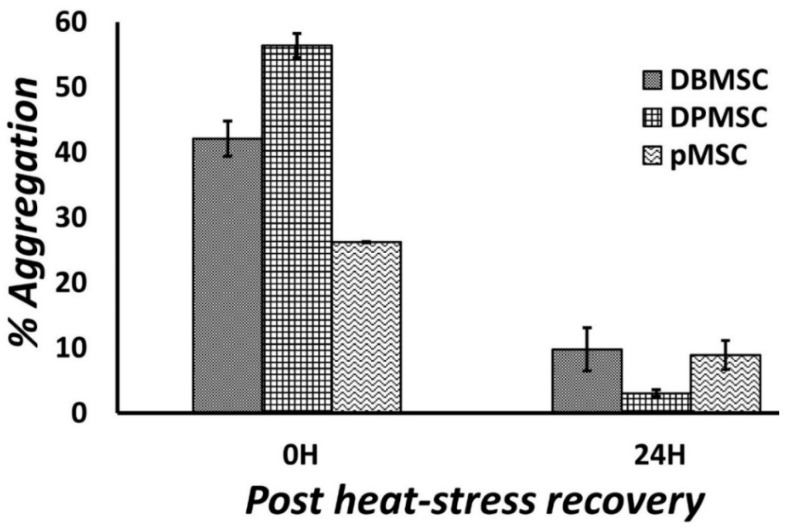
Estimation of protein aggregation in heat-stress models of DBMSCs, DPMSCs, and pMSCs. Increase in aggregation was measured as percentage change in fluorescence as compared to total proteome of cells grown at 37 °C. All the three cell types exhibited the highest presence of aggregates immediately after heat stress, i.e., recovery time 0H confirming existence of proteotoxic stress.

We estimated protein aggregation in the maximally stressed cells (i.e., those exposed for maximum duration at the highest exposure temperature) immediately at commencement of heat stress, i.e., 0H, and at 6 and 24 h post-heat stress (Figure 2). The presence of aggregates was detected immediately at 0H with the highest around 55% aggregation in DPMSCs, followed by approximately 40% in DBMSCs, and a relatively less value of around 25% in pMSCs. One reason for having less aggregation in pMSCs compared to DPMSCs and DBMSCs could be the exposure duration of two hours compared to three hours in other two cell types. In all the three cell types, the protein aggregates seem to have been resolved six hours post-heat exposure. 

**Figure 3:**
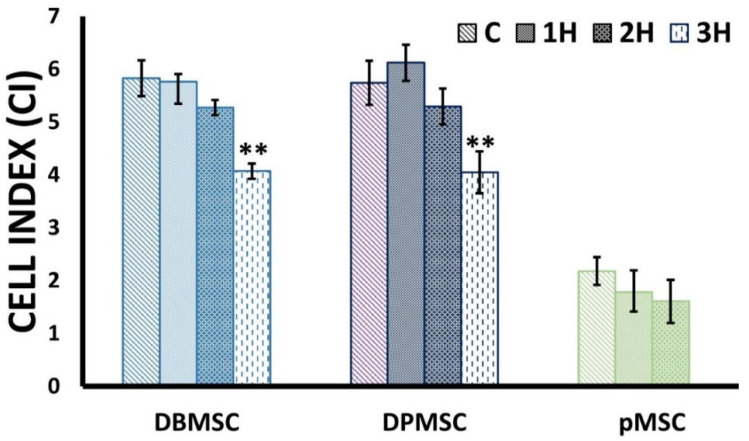
Cell proliferation assay in heat-stress models. Cells grown at 37 °C (C) and heat-exposed at 44 °C (For 1H, 2H, and 3H) were seeded in E-plate and monitored automatically. Cell Index (CI) values depicted here are 24 h post-heat stress and show statistically significant (** *p* < 0.05) reduction at 3 h exposure duration at 44 °C in DBMSCs and DPMSCs. In case of pMSCs although CI reduction of around 25% is observed, but it was not detected to be statistically significant.

We measured the impact of heat stress on cell viability and proliferation using the xCELLigence real-time cell analysis (RTCA) system. The cell behavior was monitored over a period 72 h in control cells grown at 37 °C and in cells exposed to heat at 44 °C for 1, 2, and 3 h in the case of DBMSCs, DPMSCs and 1 and 2 h in case of pMSCs. The calculated Cell Index (CI) values indicate a decrease in proliferation (Figure 3, Table 1). In the case of DBMSCs and DPMSCs, a 3-h exposure at 44 °C leads to a statistically significant (*p* < 0.05) reduction in cell proliferation, which is observable even at 24 h post-heat stress. This decreased cellular proliferation points towards the persistence of cellular stress, potentially an active heat-shock response.

### 3.2. Dose-Responsive Characteristic of Heat-Stress and Stress Response

**Figure 4:**
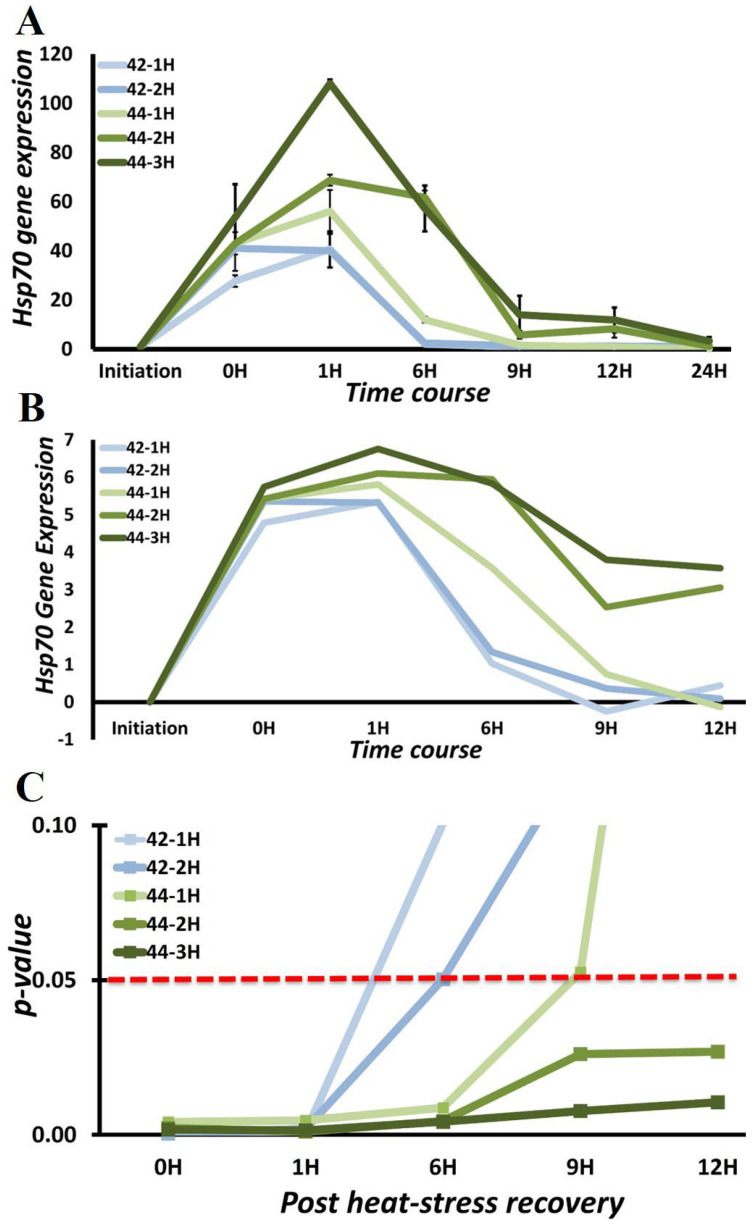
Dose-responsive nature of heat stress in DBMSCs (**A**) Time-course analysis of Hsp70 gene expression induction at 42 °C for 1 and 2 h and at 44 °C for 1, 2, and 3 h. (**B**) Log_2_ Fold Change plot for Hsp70 induction. Temperature elicits larger response as can be judged from difference between 44-2H and 44-1H. As the magnitude of heat stress increases, the heat shock response increases both in magnitude and duration as seen at 9H and 12H for 44-2H and 44-3H. (**C**) Dose responsiveness shows a pattern in statistical significance as the *p*-value varies decreases with respect to increase in magnitude of heat stress from 42-1H to 44-3H. It is further evident from *p* < 0.05 in the case of 44-2H and 44-3H up to 12 h post-heat stress.

Our aim is to mimic the proteomic stress that stem cells experience under different circumstances and study the stress response over a prolonged period. The mere existence of stress does not automatically translate into a robust and measurable stress-response. We, therefore, validated the above tested stress models for their ability to elicit a tangible and effective stress response. We measured Hsp70 (HSPA1B) gene expression immediately after heat-stress exposure and at 1, 6, 9, 12, and 24 h post heat-stress at 42 °C for 1 and 2 h and at 44 °C for 1, 2, and 3 h. We tested this approach with DBMSCs because these cells are from the maternal–fetal interface that exposes them to higher levels of circulating inflammatory factors and reactive oxygen species, consequently causing them to have high oxidative stress resistance [23]. Therefore, it is logical to validate the heat shock response in a cell type which already has an existing stress response, the heat shock response being also a function of oxidative stress [20]. Figure 4A shows the time-course analysis of Hsp70 gene expression during the recovery phase post-heat stress at 42 °C for 1 and 2 h and 44 °C for 1, 2, and 3 h. Hsp70 expression differed very marginally in the exposure temperatures of 42 and 44 °C for exposure durations of 1 and 2 h; in fact, there is almost no difference whether the cells are exposed to heat at 42 °C for 1 or 2 h (Figure 4A, Table 2). The exposure temperature of 44 °C with exposure duration of 3 h induces a measurably substantial stress response.

The dose-responsive nature of Hsp70 gene expression is evident as the exposure temperature [ET] and exposure duration [ED] increase. In the case of ET: 42 °C, the Hsp70 gene expression at 6H has come down to Log_2_ fold change of around 1, while in case of ET: 44 °C, ED: 1H, it is 3.5 and increases to more than 5.5 when ED > 1H. Although a decrease can be noticed beyond 6H in ET: 44 °C, the Log_2_ fold change is still more than 3 (Figure 4B). This difference in gene expression fold change also follows a pattern in *p*-values with the significant expression time points going from two (0H and 1H) for ET: 42 °C, ED: 1-h to five (0H, 1H, 6H, 9H, and 12H) in case of ET: 44 °C, ED: 3-h (Figure 4C). Thus, an active stress response is operational up to 1 h in low magnitude heat stress (ET: 42 °C, ED: 1-h) as compared to at least up to 12 h in high-magnitude heat stress (ET: 44 °C, ED: 3-h). Keeping our goal for comprehensive analysis of stress response in consideration, for DBMSCS, the exposure temperature (ET) of 44 °C accompanied by exposure duration (ED) of 3 h are the appropriate conditions for studying heat stress response. Utilizing similar approaches, ET: 44 °C, ED: 3 h for DPMSCs and ET: 44 °C, ED: 2 h for pMSCsfrom chorionic villi are ideal conditions for heat-stress induction.

### 3.3. Chaperone Gene Expression during Heat-Shock Response

**Figure 5:**
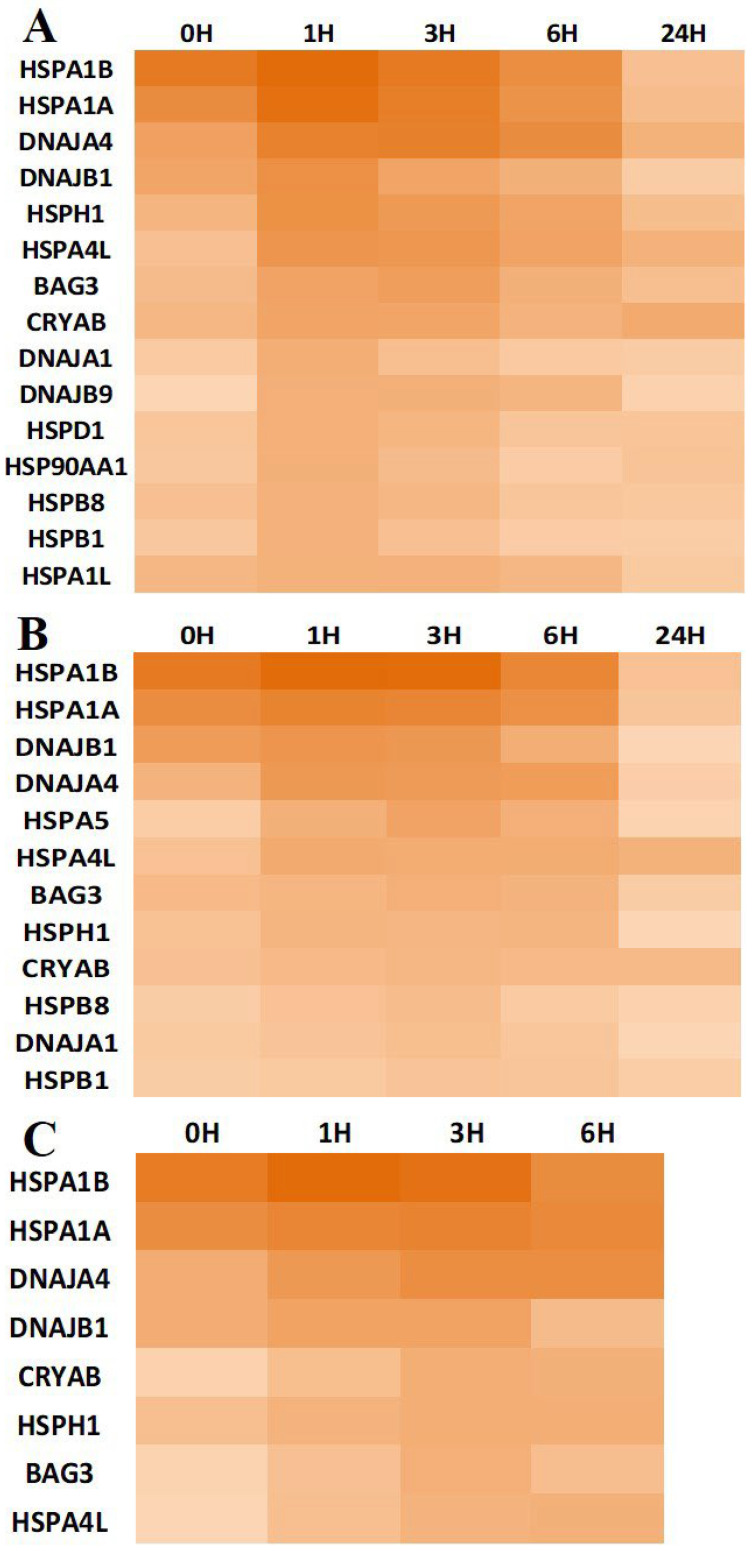
Heat maps showing temporal expression of statistically significantly (*p* < 0.05) expressed genes at conclusion of heat stress (0H) and during recovery at 37 °C at 1 h (1H), 3 h (3H), 6 h (6H), and 24 h (24H). Maximal gene-expression time point at 1H in DBMSCs (**A**), 3–6H in DPMSCs (**B**), and 3H in pMSCs (**C**). Additionally, in all the three cell types, significantly high expression of HSPA1A, HSPA1B, DNAJA4, and DNAJB1at 0H is visible. (For Fold Change values and Column Representation, see Table S2 and Figure S2).

We performed a time-dependent analysis of chaperone gene expression in our stress models of DBMSCs, DPMSCs, and pMSCs using Human Heat Shock Proteins & Chaperones RT^2^ Profiler PCR Array (Qiagen, Germantown, MD, USA), which allows for simultaneous expression analysis of 84 genes. This time-dependent chaperone gene-expression profile involved gene expression immediately after commencement of heat stress exposure (0H), and at hours 1, 3, 6, and 24 post-heat stress, when the cells were recovering at 37 °C. At 0H, we expect to identify the genes induced near-simultaneously with stress initiation. Those expressed in later stages can have a more prominent role in post-stress recovery. We set the statistically significant (*p* < 0.05) Log_2_ cut-off fold change for a gene to serve as a subject for further analyses at 1.5. For comparative analyses, this condition should be fulfilled immediately after stress exposure, i.e., 0H, or at least in one of the analyzed recovery time-point conditions. On this basis, 15 genes (Figure 5A) among heat-shock proteins and chaperones in DBMSCs, 12 genes (Figure 5B) in DPMSCs, and 08 genes (Figure 5C) in pMSCs are the overexpressed genes. In addition to these, the Heat shock 70 kDa protein 6 (HSPA6) is overexpressed at 0H (and subsequently studied points) in both DPMSCs and pMSCs as concluded from the low average threshold cycle (C_t_~16) in both as compared to C_t_ value in control samples. The exact fold change cannot be calculated reliably because of the unreliable C_t_ value (>30) in controls in both cases. The 08 genes HSPA1A, HSPA1B, HSPA4L, HSPH1, DNAJB1, DNAJB4, CRYAB, and BAG3 are common to all the three cell types and, in fact, are the only ones overexpressed in pMSCs other than HSPA6. DNAJA1, HSPB1, and HSPB8 are the common genes overexpressed in DBMSCs and DPMSCs only. In addition, HSPA5 can be detected specifically in DPMSCs, whereas HSPA1L, DNAJB9, HSP90AA1, and HSPD1 are overexpressed only in DBMSCs. Summing up, 17 out of 84 genes of the Human Heat Shock Proteins & Chaperones RT^2^ Profiler PCR Array are overexpressed altogether as part of the heat-induced proteotoxic stress response in the three placenta-derived stem cells (PDSCs). On the basis of protein family or group distribution, these 17 genes belong to eight groups: Small Heat Shock Protein, Mitochondrial 60 kDa heat shock protein, Heat shock 70 kDa protein, Heat shock protein 90 kDa alpha (cytosolic) class A, Heat shock protein 105 kDa, DnaJ homolog subfamilyA, DnaJ homolog subfamily B, and BAG family molecular chaperone regulator 3 (Table 3). Within these, six of the overexpressed genes belong to Heat shock 70 kDa protein, around 35% of the overexpressed genes, followed by four genes (around 25%) belonging to DnaJ homolog subfamilies. The other overexpressed proteins, such as J-proteins, HSPH1, and BAG3, are the ones that interact with HSPA1A and HSPA1B during their active life cycle. The ATPase reaction cycle of Hsp70s is regulated by J-proteins and nucleotide exchange factor (NEF) cochaperones by working in substrate recruitment [34,35]. Therefore, the next group of highly expressed chaperones being DnaJ homologs is not surprising. These J-proteins are part of a large family of proteins with around 40 members in humans, all of which are characterized by presence of a J-domain that binds to the N-terminal ATPase domain of Hsp70 [18,35]. Likewise heat shock protein 105 kDa also identified as HSPH1 acts a co-chaperone for HSPA1A and HSPA1B by functioning as nucleotide exchange factor [14,35,36]. Another Hsp70 family interacting protein that is overexpressed is the BAG family molecular chaperone regulator 3 (BAG3). All BAG proteins are characterized by a conserved BAG domain in their C-terminal region and BAG3 along with some other members of this family physically interact with Hsp70 potentiating a role for these proteins in Hsp70 targeting factors [37,38]. It can be thus concluded that the proteotoxic stress response in PDSCs involves overexpression of Hsp70 family proteins and to support and coordinate their activities, their interacting cochaperones and other co-factors are overexpressed along with them.

### 3.4. HSPA1A and HSPA1B Are Primary Determinants of Heat-Induced Proteotoxic Stress Response

*Primary determinants* of stress response are the genes that are substantially and significantly overexpressed *immediately after stress exposure*. This significant overexpression immediately at the onset of stress indicates their potential primary influence in the stress response. Thus, *maximal expression time point*, i.e., time-point post-heat stress at which the highest expression is attained is another factor in their identification.

**Figure 6:**
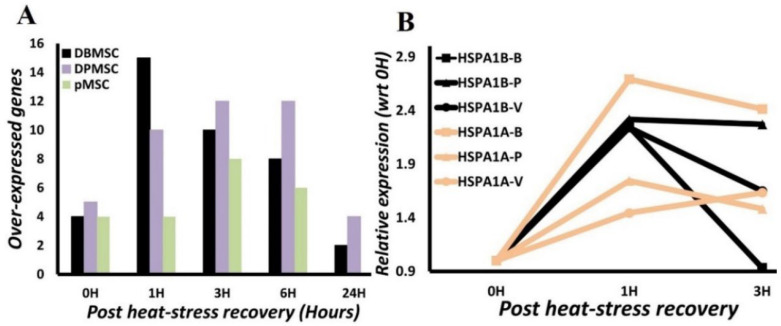
(**A**) Number of over-expressed genes. Number of genes overexpressed immediately at conclusion of heat stress (0H) and during recovery at 37 °C at 1 h (1H), 3 h (3H), 6 h (6H), and 24 h (24H). Minimum variation in number of overexpressed genes is seen at 0H with 4–5 genes overexpressed across the three stem cell types. (**B**) Variation of HSPA1A and HSPA1B gene expression in ‘post heat-stress recovery phase’. Less-that-3-fold change in gene expression with respect to expression after heat-stress exposure, i.e., 0H. B in the label represents DBMSCs, P represents DPMSCs, and V represents pMSC.

From the time-course gene expression analysis, we identified four genes in DBMSCs and pMSCs, and six in case of DPMSCs are overexpressed at 0H (Figure 6A). Four of these genes, HSPA1A, HSPA1B, DNAJA4, and DNAJB1 (Figure 5), are common to all the three cell types and are expressed significantly and predominantly at 0H. We analyzed this further by calculating the ‘recovery gene-expression fold-change’ by normalizing the calculated gene expression fold-change to 0H fold-change. This sets a value of 1 for gene-expression fold-change at 0H, and recovery phase gene-expression fold-change is analyzed in comparison to this (Figure 6B). We further set a threshold of <3 for ‘recovery phase gene-expression fold-change’ for a gene to qualify as a primary determinant of stress response. This less than three-fold change criterion is selected to have a stringent criterion for ‘recovery phase gene-expression’, while appreciating the fact that active transcription in the recovery phase is a hallmark of stress response and is expected to persist even hours after stress exposure [30]. Therefore, continued expression at 1H and beyond is not surprising, even though the expression is at low levels. Only two genes, HSPA1A and HSPA1B, from among the four early phase overexpressed genes fit this criterion of criticality with HSPA1A 1H/0H fold-change between 1.4–2.7 while in the case of HSPA1B it is between 2.2–2.3 (Figure 6B). An important point to consider is that HSPA1B and HSPA1A are the two topmost overexpressed genes among significantly overexpressed genes in Human Heat Shock Proteins & Chaperones RT^2^ Profiler PCR Array (Figure 5, Table S2), augmenting the argument in favor of their critical importance to heat-induced proteotoxic stress response. Thus, in all the three placenta-derived stem cells, the very early stages of the transcriptional response to proteotoxic stress are primarily mediated through two Hsp70 members, HSPA1A and HSPA1B.

We further analyzed the HSPA1A and HSPA1B gene expression temporal characteristics to identify their transcriptionally active period in the stress response. Highly significantly measurable HSPA1A and HSPA1B gene expression is detected immediately after heat-stress exposure: 5–7 log fold increase (HSPA1A: 5–6.2, HSPA1B: 6.7–7.2) (Figure 5, Table S2). There is an observable upward trend in gene expression at 1H with a very stable approximately two-fold change (2.2–2.3) in HSPA1B. In HSPA1A, there is variation in the fold expression with 2.7 in DBMSCs and <2 in DPMSCs and pMSCs. Nonetheless, this 1H is the ‘maximal gene-expression point’ of these genes (Figure 6B). The gene expression does not reduce substantially even up to 3H, around only 0.1–0.2-fold reduction in expression is observed in most cases. At 6 h post-heat-stress exposure, a steady decline in gene expression can be identified in all the analyzed conditions, albeit with different decline rates (Figure 5, Table S2). Irrespective of the differences in decline rates, there is a similar directionality in HSPA1A and HSPA1B gene-expression patterns in these three placenta-derived stem cells. Thus, a conclusion is drawn that there is an active transcription of HSPA1A and HSPA1B up to six hours post-heat stress exposure.

### 3.5. HSPA1A and HSPA1B Protein Expression follows a Temporal Pattern

We assessed the HSPA1A and HSPA1B protein expression using the same time-series-based approach as applied in gene expression analysis. Initially, we evaluated the protein expression immediately after heat-stress exposure, i.e., at 0H and at 1H, 3H, 6H, 9H, 12H, and 24H during recovery at 37 °C. For further analyses, we selected the time points that appeared to be relevant and significant in the protein expression process: (i) 0H, immediately after cells are relieved of stress, (ii) 1H, first hour after recovery and important from gene-expression point of view, (iii) 6H, where gene expression can be seen to be receding, and (iv) 24H, where gene-expression almost hits the pre-induction states.

**Figure 7:**
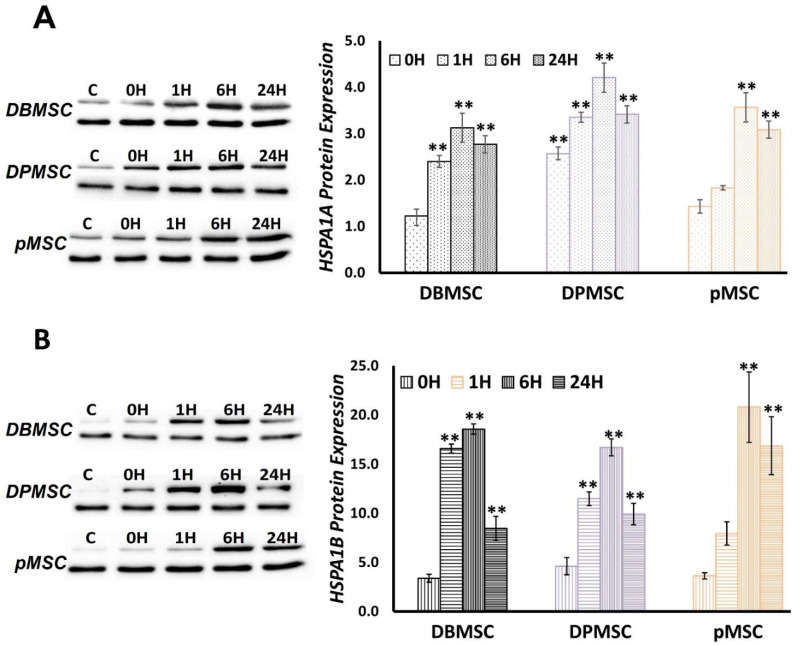
(**A**) HSPA1A and (B) HSPA1B protein expression in DBMSC, DPMSC and pMSC, immediately at commencement of heat stress (0H) and during recovery at 37 °C at 1 h (1H), 6 h (6H), and 24 h (24H). More HSPA1A (**A**) in control samples as compared to HSPA1B (**B**). Protein expression is reported to be statistically significant ** at *p*-values < 0.05.

In non-stressed control cells, i.e., cells continuously growing at 37 °C, HSPA1A protein expression is remarkable than HSPA1B. However, overall, there is a similar trend in the expression of both proteins, although with different magnitudes (Figure 7). In the case of HSPA1A the significant fold-change varies between 2 and 4.5, while for HSPA1B, it ranges from 11 to 20. In terms of temporal distribution, in all the three cell types, both HSPA1A and HSPAB1A are characterized by protein expression in the first recorded sample, i.e., immediately after heat-stress exposure (0H). At 1H, i.e., after cells have recovered at 37 °C for one hour, significant overexpression for both HSPA1A and HSPA1B can be seen in DBMSCs and DPMSCs, while in the case of pMSCs, the protein expression levels are still low at this stage. The time point of 6H post-recovery at 37 °C is the critical one from a protein-expression point of view in all the three cell types and for both proteins. Thus, 6H is the *‘maximal protein-expression point’* where statistically significant maximal protein expression is observed in all cases. The fold change in protein expression ranges between 3.1–4.2 and 16–21 for HSPA1A and HSPA1B, respectively. The protein expression starts receding after this point, but not significantly, and even at 24 h post-heat stress, protein expression fold-change ranges between 2.8–3.5 and 8–17 for HSPA1A and HSPA1B, respectively. Thus, the heat-induced proteotoxic stress response, as represented by HSPA1A and HSPA1B expression, is significantly and substantially active even 24 h post-exposure of heat stress.

### 3.6. HSPA1B Expression Dominates HSPA1A Expression: A Unified Model

**Figure 8:**
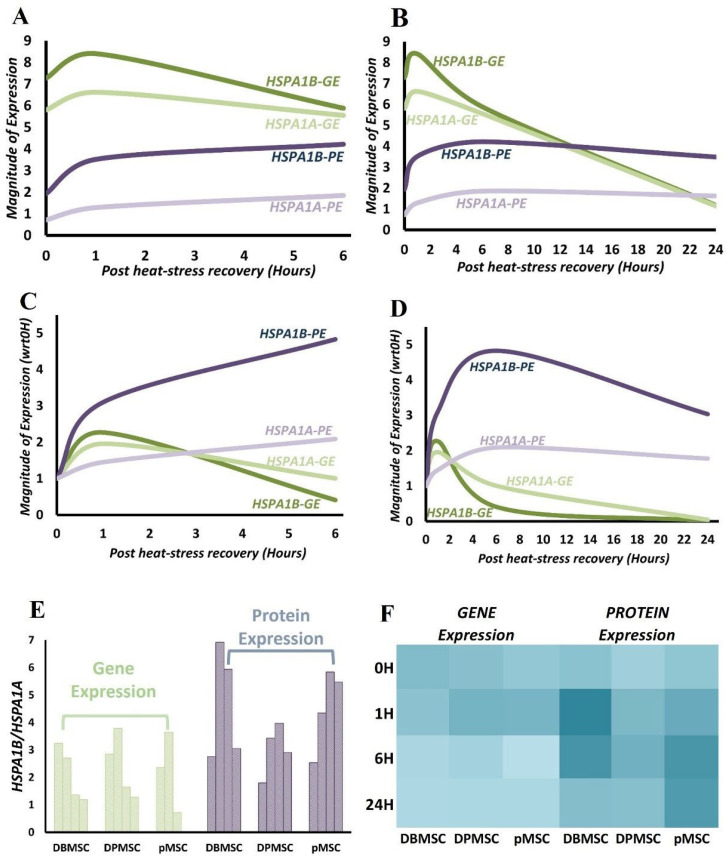
HSPA1B Expression Dominates HSPA1A Expression. (GE: Gene expression. PE: Protein expression) The time courses plotted here are from merged gene and protein expression Log_2_ data for the three PDSCs. For each cell type, the results are at least from two individual experiments. (**A**,**B**) Smoothened scatterplot of the averaged Log_2_ fold change in expression with respect to expression in control cells. (**C**,**D**) Smoothened scatterplot of the averaged fold change in expression with respect to expression at 0H. (**E**) Ratio of HSAP1B expression to HSPA1A expression. (**F**) Heat-map representation of the HSPA1B/HSPA1A ratios. HSPA1B dominance is higher in protein expression and is very well observed even 24 h post-stress exposure.

From the time-course gene- and protein-expression data analysis for HSPA1A and HSPA1B, we believe that further integrative analysis to arrive at a modular interpretation of proteotoxic stress response in PDSCs is possible. To achieve this unified model, we merged and averaged the respective data for gene and protein expression for the three cell types. This data transformation is possible because there are no large-scale differences between the expression fold-changes at the analyzed time points (Figure S3). This simplistic methodological adaptation is also made possible by the fact that we are primarily relying on relative changes of gene and protein expression, and there is a strong co-directionality in expression patterns in all the three cell types [39]. The stress response transcription–translation dynamics of HSPA1A and HSPA1B are interpreted at two levels: on transitioning of cells from (i) steady-state to expression induction, i.e., expression at 37 °C vs. expression under stress, (ii) stress phase to recovery phase, i.e., expression at 0H vs. expression post-stress recovery at 37 °C. The first scenario involves utilizing the Log_2_ expression fold change values, which are the values relative to normal cells growing at 37 °C, thus, healthy control cells serve as a reference point (Figure 8A,B, Table S3). For the second scenario, we normalized the expression values against 0H values, thus using stressed cells as the reference point, which means evaluating cells recovering at 37 °C with reference to stressed cells (Figure 8C,D, Table S4).

HSPA1B protein expression compared to HSPA1A is at least 2-fold all along, while the gene-expression ratio shows greater fluctuation between different time points (Figure 8A,B). The difference is noticeable in the mRNA consumption/decay rate for HSPA1B. The slope for HSPA1A and HSPA1B expression with respect to control starts overlapping at around 6H (Figure 8A,B), meaning the presence of similar levels of mRNA. At 1H, the HSPA1B:HSPA1A is around 3.5, reaching up to 1.25 at 6H, clearly pointing towards a higher rate of HSPA1B mRNA utilization in the earlier stages of stress response. A clearer view of the transcription dynamics during recovery phase is attained when making comparisons of recovery states with stressed state (Scenario ii proposed above) (Figure 8C,D). This represents a new steady state post-stress exposure. Here, the faster HSPA1B mRNA consumption rate post-heat stress is clearly visible in gene expression curves from the recovery phase. At 1H, post-stress translation starts to increase, but there is visible dominance of HSPA1B translation over transcription (Figure 8C). At this stage, the HSPA1B/HSPA1A is 2.7–3.8 and 3.4–6.9 for gene and protein expression, respectively (Figure 8E). It is evident that HSPA1B protein is produced at least 3-fold more than HSPA1A, as early as 1H after cells recover at 37 °C. This difference in protein expression is also visible at 6H, which is the maximal protein expression time point, and the HSPA1B/HSPA1A, at this point, varies from 3.9 to 5.9 (Figure 8E,F, Table S5). This trend is seen even 24 h after stress with HSPA1B/HSPA1A around 3 in DBMSCs and DPMSCs, while in pMSCs, it is still at levels of around 6H. Therefore, irrespective of cell-based differences, there is clearly a much higher expression of HSPA1B as compared to HSPA1A all along the stress response period of 24 h.

## 4. Discussion

Stem cell behavior warrants persistently enhanced proteome maintenance networks, the need for which becomes further compounded in their applications in therapeutics, which requires adapting to different cell physiological and differentiation states. Using a combination of *magnitude*, defined by exposure temperature [ET], and *duration*, defined by exposure duration [ED], we generated heat-induced models of proteotoxic stress in placenta-derived stem cells (PDSCs). We expect these models to simulate the proteome-stressed physiological state of stem cells. Protein structural maintenance and stability is an outcome of the intermolecular and intramolecular interactions. Anything potentially disturbing these interactions, such as temperature, oxidative stress, and heavy metals, is a proteotoxic stressor and leads to protein misfolding and potentially aggregation [20,40,41,42]. We confirmed the presence of proteotoxic stress by measuring proteome aggregation and cell proliferation, and validated the dose-response nature of stress by monitoring the Hsp70 gene expression. The time series analysis of stress and stress response detects the presence of an active stress response even 24 h post-stress exposure. It is noteworthy that stress-response pathways are transient in nature, but ensure synchronization with cell physiological state. Consequently, they are controlled tightly in both magnitude and duration, which is proportional to the severity or dose of the stress itself [20,43]. Thus, different stages of a stress-response pathway are related to different cell-fate decisions. We believe that these time-course-based proteotoxic stress models are an excellent platform to study these different phases of the stress response. Each time point potentially represents a significant component of the overall stress response.

Gene expression modulation is central to cellular adaptation to stress and pathophysiological requirements. We identify Hsp70 family chaperones (Section 3.3 and Section 3.4) as the predominant ones expressed in our proteotoxic stress models in PDSCs. In multiple instances, different heat-shock proteins are endogenously expressed in abundance in pluripotent stem cells relative to terminally differentiated cells. For example, in embryonic stem cells (ESCs), higher expression of heat-shock proteins (Hsps) and their subsequent interaction with transcription factors is essential for cell development and functioning [44,45,46]. The downregulation of Hsp70 protein 5 (HspA5) in isolated head and neck cancer stem cells (HN-CSCs) is related with reduction in self-renewal properties and inhibition of tumorigenicity [47]. Like embryonic and cancer stem cells, high HSPA5 expression has been detected in hematopoietic stem cells (HSCs) [48]. In characterization of shared and unique chaperone expression profiles in different types of stem cells, high expression levels of Hsp70 protein 5 (HspA5), Hsp70 protein 8 (HspA8), and Hop (Stip1) are reported [44,49]. Thus, the maintenance of proteome stability in stem cells is a demanding process, consequently, is accompanied by high chaperone and co-chaperone expression that act as a buffer against different stressors. To our knowledge, there is no information available regarding the expression of heat shock proteins in placenta-derived stem cells; therefore, the overexpression of HSPA1A and HSPA1B here signifies their importance in proteome maintenance under stress conditions in these cells.

Temporally, chaperones are among the first group of proteins synthesized as part of the heat-shock response, and this very well correlates with their demand in protein refolding and proteostasis restoration [20,50]. The ‘maximal gene-expression time point’ of 1 h (Section 3.4) for HSPA1A and HSPA1B is consistent with this concept that the heat-stress gene expression regulation occurs at transcription level [51,52]. Overall, for all the overexpressed chaperones in the three PDSCs, the ‘maximal gene-expression time point’ occurs between 1 and 6 h into the stress response and varies between the three cell types (Figure 5). This expression profile of PDSCs matches well with that of other systems such as Drosophila [50], *C. elegans* [53], cells such as HeLa [54], mouse embryonic fibroblasts [52], and humans [55]. Stress response is a dose-related phenomenon, and consequently, gene expression calibration is proportionate to the stress stimulation. We detected the maximum protein aggregation level at 0H (Figure 2), representing the peak proteotoxic stress level. Chaperones have a primary role in maintaining protein folding states and are expressed in stoichiometric amounts in proportion to the aberrantly folded proteins. Although the ‘maximal protein-expression time point’ is observed 6 h after the cells are relieved of heat stress (Section 2.5), there is no lag between gene and protein expression as the protein expression can be measured immediately after heat stress, i.e., 0H (Figure 7).

However, there is a vast difference in the amounts of mRNA and protein at this point, characterized by the presence of significantly transcribing mRNA (Figure 8A). In the first hour of recovery, both mRNA and protein keep increasing in a homodirectional manner at a comparatively rapid rate (Figure 8A and Figure 7B). This initial spiking in the mRNA is observed in other stress proteins such as Trafd1 in LPS-stimulated mouse dendritic cells [56], in heat shock proteins in the case of rapamycin challenge to yeast [57], and in HSPA5/GRP78 in the ER stress model in HeLa cells [54]. Stress response gene expression has a characteristic ‘transcriptional burst’ pattern; a hyper-activated state ensuring time-bound cooperation among gene expression machinery constituents to facilitate a proportionate stress response [51,58,59]. As a primary response to stress, there is an immediate repression of transcription, as early as within 10 min of stress exposure, whereas heat-induced transcription activation is observed as early as 2.5 min [51]. This transcriptional redirecting is a function of multiple regulatory events that include chromatin remodeller action at promoter and along the gene, transcription factor recruitment dynamics, promoter proximal PolII pausing establishment and maintenance, and release of paused PolII into productive elongation [51,60]. The transcriptional activation of heat-shock protein genes is coordinated through heat shock factor-1 (HSF1) by its binding to the cis-acting sequences known as heat shock elements (HSEs), the binding being dependent on trimeric HSF1 [6,61,62]. In non-stressed cells, HSF1 exists as inactive monomer in complex with chaperones Hsp70 [63] and Hsp90 [64]. According to the chaperone titration model, the non-native proteins titrate these chaperones away from HSF1, allowing it to trimerize and induce heat-shock protein gene transcription [61,62]. Simultaneously with the induction of chaperones and other proteostasis network members, there is a protein synthesis shutdown, which minimizes the production of fresh substrates for proteostasis. As the proteotoxic stress ceases and adequate free chaperone capacity is reinstituted, the HSF1 binding partners, viz., Hsp70 and Hsp90, rebind to restore the balance [65]. However, transcription induction does not automatically lead to an immediate increase in protein levels because RNA processing, such as maturation and export, requires some time along with the translation, hence, the poor mRNA–protein correlations at this stage [66,67]. However, to counter the demand for stress response proteins, mechanisms such as bypassing mRNA quality control are in place, which ensures minimal lag between transcription and translation [68]. Additionally, stress response pathways are antagonistic to growth-related programs and have different transcription regulating mechanisms, such as transcription-facilitative gene-structural organization and nucleosome reorganization apparats [60,69]. Pertinent to the Hsp70s, Hsp70 proteins have a self-mRNA stabilization ability and, in Trypanosoma, has been observed during heat shock [70]. In the DTT-induced ER stress model in HeLa cells GRP78, an important ER stress response factor, the down-regulation of mRNA expression and upregulation of protein expression sets in at the 16-h time point; a relatively high expression of both gene and protein ascribed to its stress phase importance [54]. Despite the translational predominance observed at 6 h post-heat stress, the continued gene expression at substantial levels points toward consonance between gene and protein expression. Thus, the period of up to six hours post-stress exposure is transcriptionally and translationally active for HSPA1A and HSPA1B. At 24 h, the measurable gene expression levels have effectively reached pre-stress induction levels, and protein levels are significantly high (Figure 8D). Irrespective of the mechanisms that direct the presence of higher mRNA levels or their trafficking to translation, proteotoxic stress HSPA1A and HSPA1B gene expression in PDSCs is to a large extent homologous to stress-response behavior in other systems. The early protein expression signifies their requirement in the stress response. The ‘maximal protein-expression time-point’ at 6 h potentially represents an essential stage in the proteotoxic stress response pathway, and continued protein expression at 24 h highlights their relevance to the broad proteotoxic stress response.

The palpable differences in HSPA1B protein levels (Section 3.6) compared to HSPA1A highlight a differentially significant role of HSPA1B. HSPA1A and HSPA1B share more than 99% protein identity and differ in their amino acid composition at only two positions, 110 and 499. In HSPA1A, these positions are occupied by glutamic acid and asparagine, respectively, whereas in HSPA1B, aspartic acid and serine occupy the corresponding positions [31]. They belong to a large family of Hsp70 homologs (Table S6) expressed in a compartment-specific manner with expression levels regulated according to cellular requirements [15]. Despite this high homology, Hsp70s have a specialized functional landscape [22,23]. This is due to their ability to interact with a diverse network of proteins such as members of the J-domain protein (JDP) family [20], nucleotide exchange factors (NEFs) [15,48], chaperones such as small heat-shock proteins and chaperonins [15,49], and other proteins that play a role in defining their functions. This operational and functional diversity necessitates their precise identification and characterization.

Through our stress models presented here, we outline a platform for detailed analysis of proteotoxic stress response in PDSCs and report differential relevance of HSPA1A and HSPA1B. Their functioning is not static, and it fluctuates in response to the cell’s physiological state; therefore, future studies should identify the respective interaction partners and homolog-specific regulatory networks. This is relevant not only in enhancing the understanding of Hsp70 role in stem cell biology, but also in its application in augmentation of stem-cell therapeutic applications where heat-shock pre-conditioning as a modulation tool in cell stabilization is used, albeit with some adverse outcomes [71,72,73]. With a better knowledge and understanding of stem cell maintenance and stress-response pathways, targeted approaches can be developed to enhance the viability and behavior of transplanted stem cells.

## Figures and Tables

**Table 1:** Cell proliferation analysis in heat-stress models. Percentage Difference in Cell Index (CI) between heat-stressed cells and control cells (grown at 37 °C). These values were calculated over CI values estimated at 24 h post-heat stress.

Cell Type	Exposure Duration [ED]	% Difference Cell Index (CI)
DBMSCs	1 h	1.13
	2 h	9.45
	3 h	30.15
DPMSCs	1 h	−6.61
	2 h	7.74
	3 h	29.48
pMSCs	1 h	18.28
	2 h	26.40

**Table 2:** Heat-stress model of DBMSC. Hsp70 gene expression fold-change values from five circumstance conditions involving a combination of two exposure temperatures (ET) and three exposure durations (ED).

Exposure Temperature [ET]	Exposure Duration [ED]	Hsp70 Induction
42 °C	1 h	40.67 ± 7.7
	2 h	41.04 ± 6.3
44 °C	1 h	56.07 ± 8.4
	2 h	68.77 ± 2.38
	3 h	108.13 ± 1.54

**Table 3:** Classification of overexpressed genes according to Heat Shock Protein or Chaperone.

Chaperone Family	Overexpressed Genes	Proportion
Small Heat Shock Protein	HSPB1, HSPB8, CRYAB	3/8
60 kDa heat shock protein, mitochondrial	HSPD1	1/1
Heat shock 70 kDa protein	HSPA1A, HSPA1B, HSPA1L, HSPA4L, HSPA5, HSPA6	6/11
Heat shock protein 90 kDa alpha (cytosolic), class A	HSP90AA1	1/1
Heat shock protein 105 kDa	HSPH1	1/1
DnaJ homolog subfamilyA	DNAJA1, DNAJA4	2/4
DnaJ homolog subfamily B	DNAJB1, DNAJB9	2/11
BAG family molecular chaperone regulator 3	BAG3	1/1

## Supplementary Materials

The following supporting information can be downloaded at: https://www.mdpi.com/article/10.3390/cimb44100324/s1. Table S1: Gene List for Human Heat Shock Proteins & Chaperones RT^2^ Profiler PCR Array. Table S2: RT^2^ Profiler PCR Array results. Table S3: The gene and protein expression fold change in comparison to control cells. Table S4: The gene and protein expression fold change in comparison to 0H. Table S5: HSPA1B/HSPA1A Ratios. Table S6: The human Hsp70 family of chaperones. Only the differentiating traits are highlighted. Considerable input from [16]. Figure S1: Cell culture and heat-stress experiments in Placenta-derived stem cells. Figure S2: Significantly expressed genes from the Human Heat Shock Proteins & Chaperones gene expression profile at conclusion of heat-stress. change values in comparison to 0H. Figure S3: Time course of gene and protein expression.
