# Supplementary material for: Heat-Induced Proteotoxic Stress Response in Placenta-Derived Stem Cells (PDSCs) Is Mediated through HSPA1A and HSPA1B with a Potential Higher Role for HSPA1B
Source: Curr Issues Mol Biol. 2022 Oct 10;44(10):4748–68. doi: 10.3390/cimb44100324 (PMC9600182; doi:10.3390/cimb44100324)
Supplement: Supplementary file 1 [file cimb-44-00324-s001.zip › Figures S1-S3.pdf]

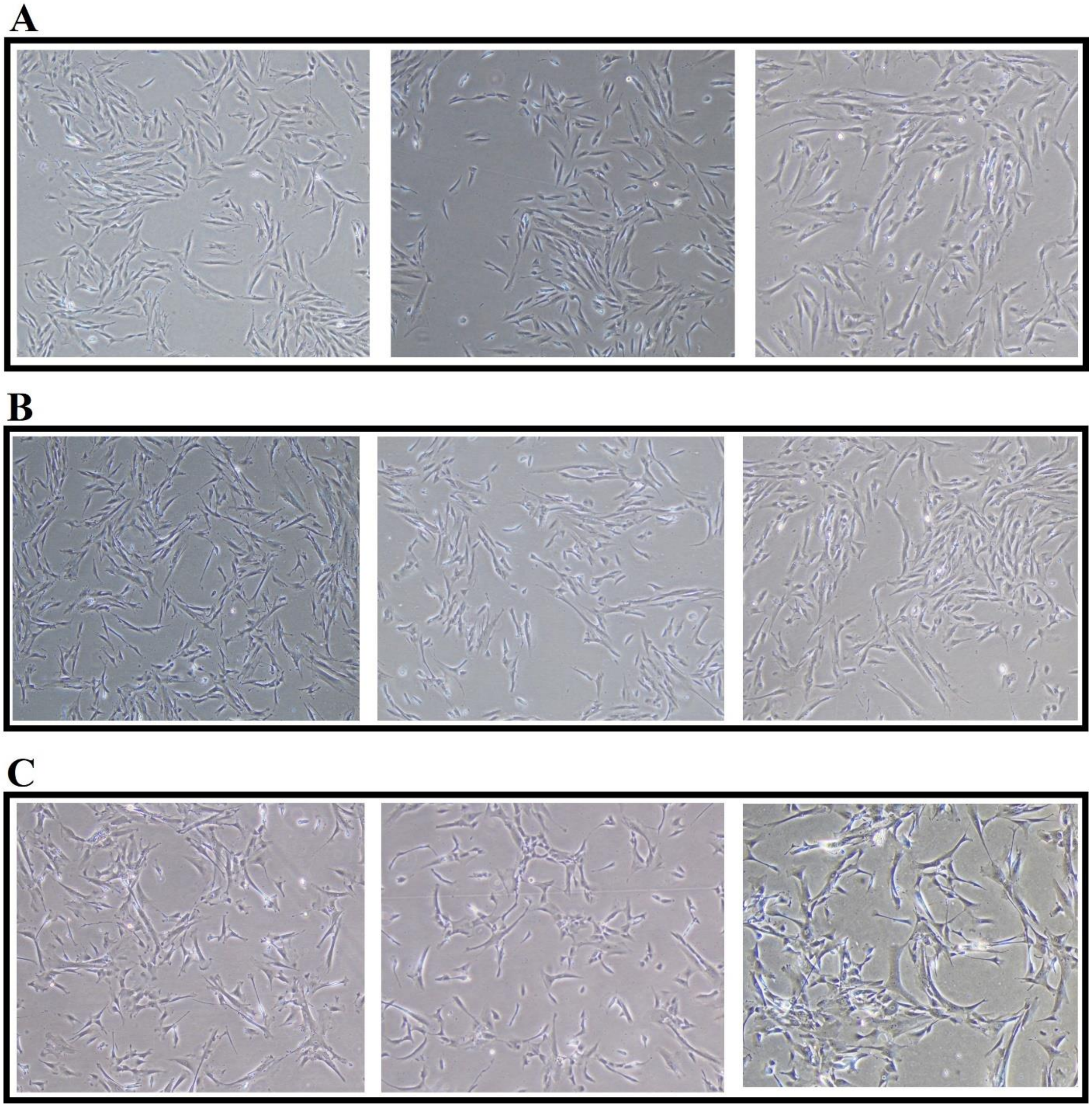

Figure S1. Cell culture and heat-stress experiments in Placenta-derived stem cells. (A) DBMSC (B) DPMSC , and (C) pMSC. Figures on the Left is Control cells, Middle is 0H i.e., immediately after heat-stress, and Right is cells recovered 24-hours post heat-stress exposure.

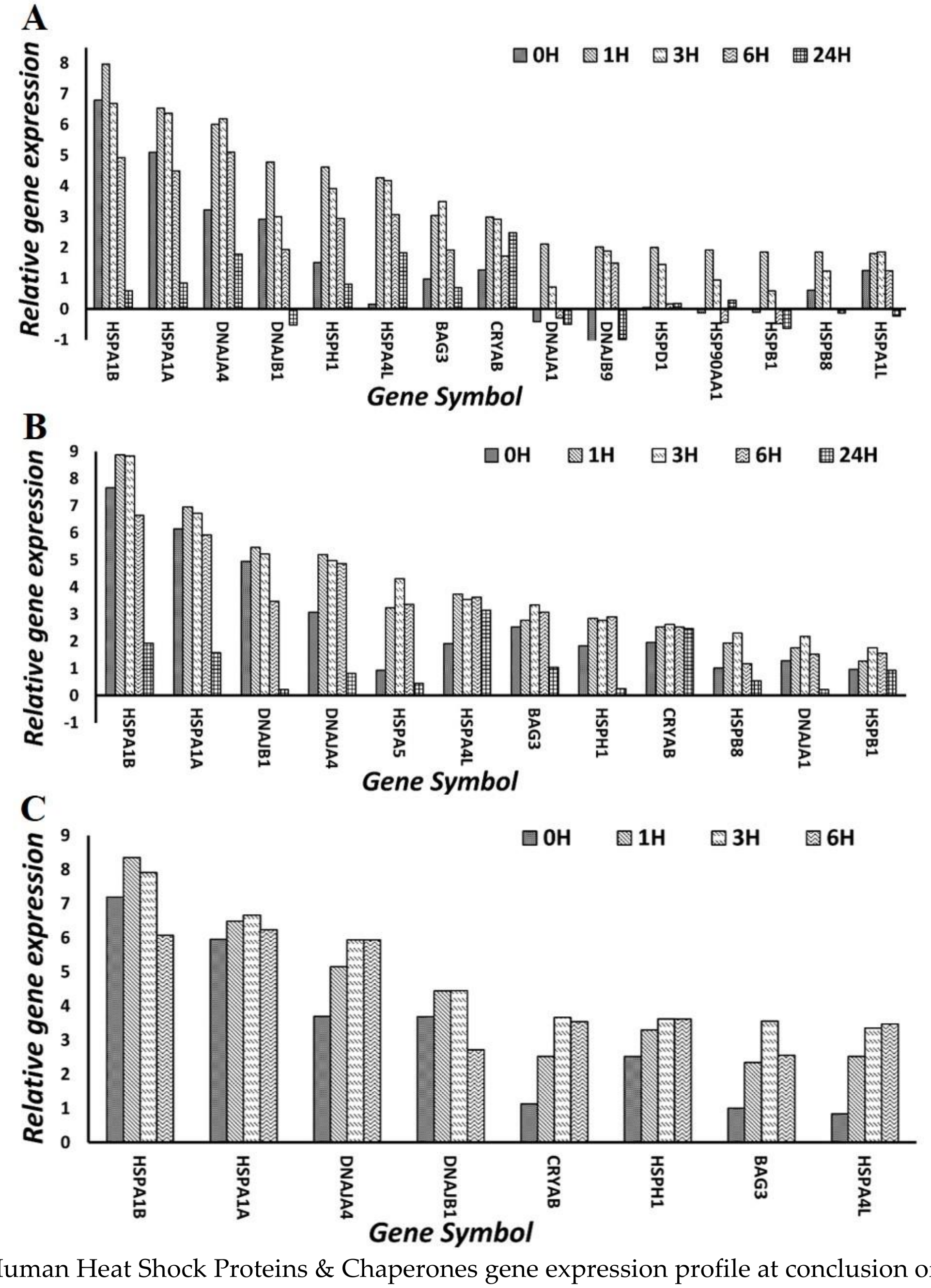

Figure S2. Human Heat Shock Proteins & Chaperones gene expression profile at conclusion of heat-stress (0H) and during recovery at 37˚C at 1-hour (1H), 3 hours (3H), 6 hours (6H), and 24 hours (24H). (A)15 genes in DBMSCs, (B)12 genes in DPMSCS, and (C) 8 genes in pMSCs were detected to be overexpressed in a statistically significant ($p<0.05$) manner with Log_2 fold-change of 1.5, in at least at one of the analyzed time-points.

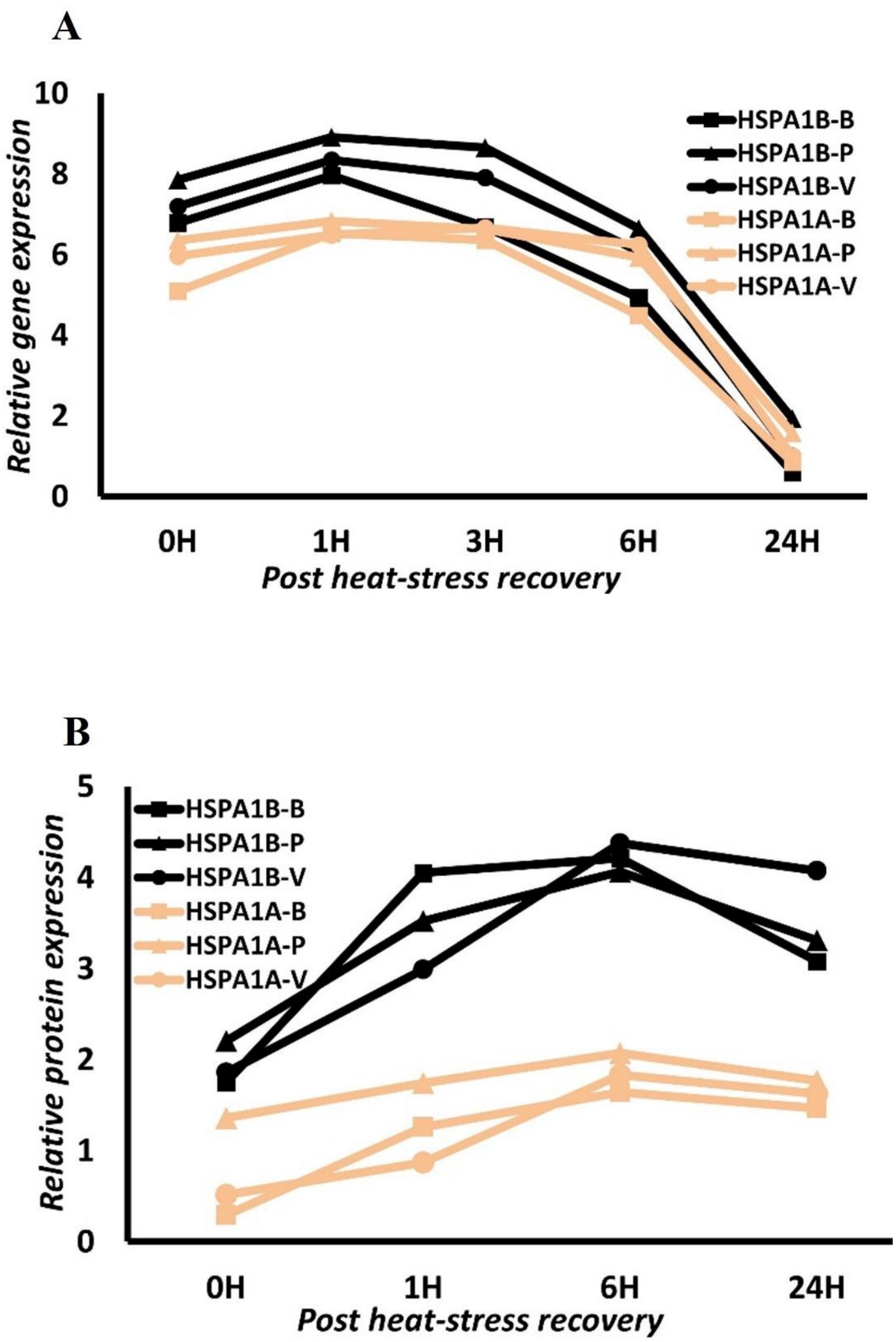

Figure S3: (A) Time-course of gene-expression. (B) Time-course of protein-expression. Log_2 fold-change values as function of time are plotted over 24 hours
